# A Data-Efficient Machine Learning Approach for Breast Ultrasound Lesion Classification Integrating Image-Derived Features and Sonographic Descriptors

**DOI:** 10.3390/diagnostics16050664

**Published:** 2026-02-25

**Authors:** Adil Gursel Karacor, Sevim Sahin

**Affiliations:** 1 Department of Industrial Engineering, Faculty of Engineering and Natural Sciences, Fenerbahce University, Istanbul 34758, Turkey; adil.karacor@fbu.edu.tr; 2 Department of Electrical and Electronics Engineering, Faculty of Engineering and Natural Sciences, Fenerbahce University, Istanbul 34758, Turkey

**Keywords:** breast ultrasound, diagnostic decision support, lesion classification, sonographic descriptors, feature fusion, small datasets

## Abstract

**Background/Objectives:** Breast ultrasound is widely used for the diagnostic evaluation of breast lesions; however, reliable lesion characterization remains challenging due to substantial image heterogeneity and the limited size of most clinically available datasets. These constraints reduce the generalizability of end-to-end deep learning approaches in routine practice. The objective of this study was to evaluate a data-efficient diagnostic framework that integrates image-derived features with clinical sonographic descriptors to improve breast ultrasound lesion classification in small cohorts. **Methods:** Ultrasound images from the publicly available BrEaST-Lesions dataset were processed using a pretrained convolutional neural network to extract compact image feature representations from full images, lesion masks, and cropped tumor regions. These features were combined with manually recorded sonographic descriptors after label encoding to form a unified tabular dataset. Gradient-boosted tree models were trained using descriptor-only and fused feature sets with fivefold stratified cross-validation and evaluated on an independent external hold-out test set. **Results:** Using sonographic descriptors alone, the best-performing model (LightGBM) achieved an external validation accuracy of 0.88, with an area under the receiver operating characteristic curve (AUC) of 0.95. Incorporation of image-derived features improved diagnostic performance on the external test set, yielding an accuracy of 0.88, an AUC of 0.96, and a sensitivity of 1.00 for malignant lesion detection. The fused framework demonstrated more stable generalization than descriptor-only models, particularly for malignant cases. **Conclusions:** Combining image-derived features with clinical sonographic descriptors within a tabular learning framework provides a robust and data-efficient approach for breast ultrasound-based lesion classification. This strategy supports diagnostic decision-making in small ultrasound datasets and represents a clinically realistic alternative when large-scale deep learning models are impractical.

## 1. Introduction

Breast cancer remains one of the most common malignancies among women worldwide, and early detection plays a critical role in improving patient outcomes [1]. Ultrasound (US) imaging is widely used in breast cancer assessment due to its low cost, real-time capability, absence of ionizing radiation, and suitability for dense breast tissue. In routine clinical practice, breast US is frequently employed as a complementary modality to mammography and magnetic resonance imaging, particularly for lesion characterization and biopsy guidance [2]. Despite these advantages, accurate interpretation of breast US images remains challenging, even for experienced radiologists.

One of the main difficulties in breast US analysis arises from the intrinsic heterogeneity of US images. Image appearance is highly dependent on operator skill, probe positioning, acquisition settings, and patient-specific factors. Lesion boundaries may be poorly defined, and benign and malignant masses often exhibit overlapping visual characteristics. As a result, US interpretation is subject to considerable inter- and intra-observer variability, which can lead to false-positive findings, unnecessary biopsies, or missed malignancies. These challenges are further compounded by the fact that most clinical US datasets are relatively small, limiting the effectiveness of data-hungry computational approaches [3,4].

In recent years, artificial intelligence (AI) and deep learning (DL) techniques have been increasingly explored to assist breast US lesion classification. Convolutional neural networks (CNNs) have demonstrated promising performance in distinguishing benign from malignant lesions by learning hierarchical image representations directly from pixel data. However, most existing approaches rely on end-to-end deep learning models trained for classification, which typically require large, well-annotated datasets to generalize reliably. In small and heterogeneous US cohorts, such models are prone to overfitting and often behave as black boxes, offering limited interpretability and reduced clinical trust.

Parallel to advances in deep learning, tabular machine learning methods—particularly gradient-boosted decision tree ensembles—have consistently shown strong performance on structured data, especially in limited-sample settings. In breast imaging, clinically recorded sonographic descriptors such as lesion shape, margin, calcifications, echogenicity, and posterior acoustic features encode expert knowledge accumulated over decades of radiological practice. These descriptors remain highly informative for diagnosis, yet they are rarely integrated in a principled manner with modern image-based representations learned by deep neural networks [5,6,7,8]. Hybrid approaches that combine CNN-derived features with handcrafted or structured features have also been investigated in breast cancer image analysis, where such fusion strategies have been shown to improve classification performance in histopathological datasets [9,10].

Motivated by these observations, this study proposes a hybrid, data-efficient framework that converts breast US images into structured tabular representations. Instead of training a deep neural network end-to-end, we employ a pretrained ConvNeXt Tiny model as a fixed feature extractor to generate compact image embeddings. These embeddings capture high-level texture and structural information from US images while avoiding the overfitting risks associated with full network fine-tuning on small datasets. The extracted embeddings are then fused with manually recorded sonographic descriptors to form a unified tabular feature space.

Within this tabular framework, gradient-boosted tree ensembles are employed for lesion classification, leveraging their robustness, interpretability, and strong performance on heterogeneous feature sets. In addition to evaluating descriptor-only and fused descriptor-embedding models, a random feature subset search is performed to identify compact and interpretable combinations of clinical descriptors and embedding dimensions that preserve diagnostic performance. All models are assessed using stratified cross-validation (CV) and an external hold-out validation set to emphasize generalization rather than internal performance alone.

The main contributions of this work can be summarized as follows:

- An image-to-tabular learning strategy is proposed to transform breast US images into structured representations suitable for small-sample classification.
- The integration of pretrained image embeddings with clinically recorded sonographic descriptors is investigated within a unified tabular framework.
- Compact and clinically interpretable feature subsets are explored using gradient-boosted models.

Together, these contributions aim to provide a practical and transparent alternative to end-to-end deep learning approaches for breast US analysis in data-limited clinical settings.

## 2. Related Work

Breast cancer diagnosis has been the subject of extensive investigation over the past decades, particularly with the growing integration of machine learning and deep learning techniques into medical imaging. A wide range of imaging modalities, including ultrasound, mammography, and magnetic resonance imaging, have been explored for computer-aided diagnosis. Earlier systems were largely based on handcrafted radiomic or texture features that were subsequently analyzed using conventional classifiers. More recently, however, CNNs have enabled automated feature extraction directly from image data, allowing models to capture complex visual patterns that are difficult to represent using manually engineered features, and this shift has led to noticeable improvements in lesion classification performance across several breast imaging datasets [5,11,12,13].

Among the available modalities, breast ultrasound has attracted particular interest because of its accessibility, lack of ionizing radiation, and effectiveness in evaluating dense breast tissue, where mammography may be less sensitive. A number of studies have therefore focused on applying transfer learning and fine-tuned CNN architectures to ultrasound images in order to distinguish between benign and malignant lesions, often reporting encouraging diagnostic accuracy [11,12,13,14]. In addition to purely image-based models, some researchers have attempted to incorporate clinical variables or structured sonographic descriptors together with image-derived features, recognizing that these complementary sources of information may contribute to more robust and interpretable predictions [8,15]. Investigations conducted on publicly available ultrasound datasets, including BrEaST-Lesions and BUSI datasets and similar collections, have demonstrated the feasibility of such approaches, although many of these studies still face challenges related to moderate dataset sizes, variations in preprocessing pipelines, and limited integration of heterogeneous feature types, all of which can affect generalizability across patient populations [16,17].

Beyond ultrasound imaging, recent work has increasingly explored more complex neural network architectures, particularly transformer-based and hybrid deep learning models, for breast cancer diagnosis. Variants of self-attention transformer encoders, as well as hybrid convolutional–transformer frameworks, have been proposed for classification tasks using full-field digital mammography and other X-ray breast imaging data, where they have shown promising capability in capturing long-range contextual information and improving feature representation [18,19]. Other studies have investigated explainable and federated learning strategies designed to combine imaging findings with clinical risk factors, with the aim of improving both model interpretability and generalizability across institutions [20]. In addition, multimodal computer-aided diagnosis systems that jointly analyze mammography and ultrasound images have demonstrated that integrating complementary imaging modalities may enhance diagnostic reliability in certain clinical scenarios [21].

Despite the progress achieved in these areas, a considerable portion of the literature continues to rely primarily on image-derived features within end-to-end deep neural network architectures, without fully exploring alternative modeling paradigms. In particular, limited attention has been given to transforming image representations into a structured tabular format that enables the use of tree-based ensemble methods, which are often more robust in moderate-sized clinical datasets. Such an approach allows pretrained deep embeddings to be leveraged while benefiting from the stability, data efficiency, and interpretability of gradient-boosted decision trees. For this reason, the present study investigates a hybrid strategy that converts CNN-derived image embeddings into a unified tabular representation and integrates them with structured sonographic and clinical descriptors. This design enables the application of powerful boosted tree models in a multimodal setting, aiming to maintain competitive performance under small-cohort constraints while preserving model transparency and flexibility.

## 3. Materials and Methods

### 3.1. Dataset

The BrEaST-Lesions US collection contains two-dimensional US images and corresponding tumor masks for benign and malignant lesions. The dataset is publicly available and was accessed via The Cancer Imaging Archive (TCIA) [16]. The original dataset consisted of 256 cases. However, four cases were excluded due to missing or incomplete lesion mask annotations. As a result, a total of 252 cases were included in the final analysis.

**Figure 1:**
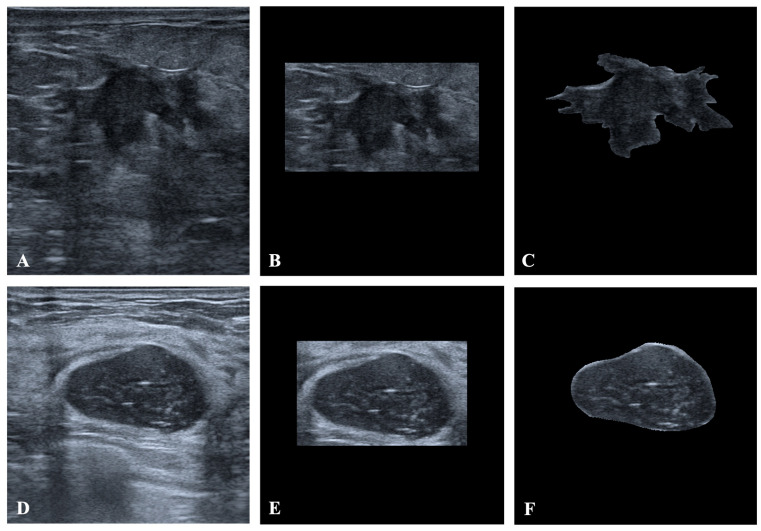
Representative examples of the three image variants generated for each case. For two different patients, full US images (**A**,**D**), cropped images tightly enclosing the lesion with a 10% margin (**B**,**E**), and masked images retaining only the pixels inside the tumor mask (**C**,**F**) are shown. The top row corresponds to one patient and the bottom row to another. For each patient, all image variants are displayed at the same spatial scale. Labels were binary (0 = benign, 1 = malignant).

Each case includes a grayscale US image and an associated binary mask indicating the lesion. The final dataset of 252 cases was deterministically split into training (approximately 80%) and external validation (approximately 20%) sets using a fixed random seed (Table 1). Stratification ensured similar benign/malignant ratios across splits. Three image variants were generated for each case: full images (original US), masked images where only the pixels inside the tumor mask are kept, and cropped images that tightly crop the lesion region with a 10% margin around the mask. Representative examples of these three image variants for two different patients are shown in Figure 1. Labels were binary (0 = benign, 1 = malignant). No data augmentation was applied; all reported image counts correspond to the original images after preprocessing and splitting.

### 3.2. Image Preprocessing

Preprocessing involved the transformation of raw ultrasound scans into the RGB color space to ensure compatibility with the pretrained architectural requirements of the feature extractor and resizing to (336) pixels, matching the input size of the ConvNeXt Tiny model. A transformation pipeline from the timm library performed normalization and augmentation suitable for inference. For masked and cropped variants, the binary mask was applied so that only the tumor pixels contributed to the embedding. Cropping expanded the mask by 10% (minimum 8 pixels) in both axes and ignored cases without valid masks. Embeddings for the full, masked, and cropped versions were saved into separate CSV files (e.g., usg_full.csv, usg_masked.csv, and usg_crop.csv) along with metadata such as case_id, file path, and split. For the modeling results reported in Table 2 and Table 3, we used embeddings extracted from the masked images only, as this variant provided the most consistent performance in preliminary experiments. The full and cropped variants were evaluated during early exploration but were not used in the final modeling pipeline. No additional ultrasound-specific intensity standardization was applied; images were processed using the default ImageNet normalization pipeline associated with the pretrained ConvNeXt backbone.

### 3.3. ConvNeXt Embedding Extraction

ConvNeXt Tiny is a modern convolutional architecture inspired by the design principles of Vision Transformers while retaining the computational advantages of CNNs. Originally introduced by Liu et al. (2022) [22], ConvNeXt employs large-kernel convolutions, inverted bottleneck blocks, and LayerNorm-based normalization to match or surpass state-of-the-art transformer-based models on ImageNet. Subsequent follow-up work has further demonstrated the competitiveness of ConvNeXt-style architectures against transformer-based models under modern training regimes [23]. Its lightweight Tiny variant (~28 M parameters) offers strong representational capacity with relatively low computational cost, making it especially suitable for medical-imaging scenarios where training data are limited.

Feature embeddings were extracted using a pretrained ConvNeXt-Tiny model implemented via the PyTorch2.8.0 (CUDA 12.8) Image Models (timm) library, developed by Wightman in 2019 [24]. In this study, ConvNeXt Tiny pretrained on ImageNet was used purely as a frozen feature extractor. The classification head was removed, leaving only the convolutional backbone and the global average pooling layer. Each preprocessed US image (full, masked, or cropped) was forwarded through this backbone to produce a fixed-length embedding vector of D = 768 dimensions, corresponding to the number of output channels in the final pooled feature map. These embeddings encode high-level semantic patterns (e.g., texture, echogenicity, and lesion structure) and were subsequently concatenated with the manually recorded sonographic descriptors to form the unified tabular feature matrix used for classification.

### 3.4. Sonographic Descriptor Processing

Clinical descriptors collected during the US examination (e.g., calcifications, halo sign, margin, shape, posterior features, echogenicity, tissue composition, skin thickening, signs, symptoms, and age) were encoded numerically. Categorical descriptors were converted to integers using label encoding (hence the suffix _LE in the feature names). Continuous descriptors, such as age and pixel size, were retained as numeric values. No additional feature removal criteria (e.g., low variance or missingness filtering) were applied beyond excluding descriptors that were absent in the dataset or not recorded consistently. A unified numeric representation was also preferred to ensure consistency across CatBoost, XGBoost, and LightGBM implementations.

### 3.5. Tabular Fusion Pipeline

After descriptor encoding, the dataset consisted of two feature groups:

(i) High-dimensional image embeddings (emb_0 … emb_767, 768 features);
(ii) Low-dimensional clinical descriptors (*_LE, Age, Pixel_size).

These were concatenated horizontally to form a single tabular feature matrix:

X ∈ ℝ^nxp^

where

- n = number of cases;
- p = total number of features (descriptors + embeddings).

The target vector of binary class labels was defined as

y ∈ {0, 1}^n^,

with 0 = benign and 1 = malignant.

No missing values were present after encoding.

Concatenation was chosen as a simple and reproducible fusion strategy suitable for small cohorts. For tree-based ensembles, feature ordering does not affect learning, and cross-feature interactions between descriptors and embedding dimensions are captured through tree splits. More complex learned fusion operators were intentionally avoided to reduce overfitting risk and to preserve interpretability.

In total, the fused feature space consisted of 780 predictors, including 768 image embedding dimensions and 12 encoded clinical descriptors.

### 3.6. Gradient-Boosted Models

Three gradient-boosted tree algorithms were used: CatBoost, XGBoost [25], and LightGBM. Each model was configured for binary classification. A small hyperparameter grid tuned the number of trees/iterations, tree depth, and learning rate. Fivefold stratified CV was performed on the training portion, and the best hyperparameters were selected using random search (20 iterations for CatBoost and LightGBM; 20 random combinations for XGBoost). For the CatBoost and LightGBM models, the search was implemented with RandomizedSearchCV. For XGBoost, a custom manual search evaluated random hyperparameter combinations and selected the one yielding the highest cross-validated AUC. After tuning, each model was refitted on the full training data and evaluated on the external validation set. Evaluation metrics included accuracy, macro-F1, area under the receiver operating characteristic (ROC) curve (AUC), malignant recall (sensitivity), and benign specificity. Let the confusion matrix entries be the following:

- TP = true positives (malignant correctly classified);
- TN = true negatives (benign correctly classified);
- FP = false positives (benign misclassified as malignant);
- FN = false negatives (malignant misclassified as benign);

The metrics were computed as follows:

- Accuracy:

Accuracy = (TP + TN)/(TP + TN + FP + FN)

- Malignant recall (sensitivity):

Recall_1_ = TP/(TP + FN)

- Benign specificity:

Specificity_0_ = TN/(TN + FP)

- Macro-F1:

Computed as the arithmetic mean of class-wise F1 scores, where

F1 = 2 × (Precision × Recall)/(Precision + Recall)

and precision/recall is computed separately for each class.

- AUC:

Computed from the class-1 predicted probabilities using the ROC curve.

### 3.7. Random Feature Subset Search (500 Trials)

To explore whether a smaller and more interpretable set of predictors could be identified, we conducted a random feature subset search. From the full pool of numeric features in the fused matrix, 500 subsets of size k = 10 were sampled without replacement. For each subset, a CatBoost classifier was trained using light randomized hyperparameter tuning, and its performance was estimated using a fivefold CV. The subsets were ranked according to their cross-validated performance, and the most consistently informative combination was selected. This procedure ultimately yielded a compact final set of 10 features that balanced accuracy, stability, and interpretability.

Subsets were ranked based on their average cross-validated performance on the training data (using the same evaluation metrics reported in the manuscript), and the most stable top-ranked subset was selected solely from the cross-validation results. All subset ranking and hyperparameter tuning steps were conducted exclusively within the training portion using fivefold cross-validation. The hold-out validation set was never accessed during subset search or model selection and was used only once for final evaluation. This protocol prevents selection bias and ensures that the reported hold-out results represent an unbiased estimate of external performance.

### 3.8. Model Interpretability (SHAP)

To strengthen interpretability, we employed SHAP (SHapley Additive exPlanations) to analyze the CatBoost classifier on the untouched external validation set. Global feature importance was quantified using mean absolute SHAP values and visualized through a SHAP summary bar plot and a beeswarm plot. To provide case-level interpretability, SHAP waterfall plots were generated for representative samples, illustrating how handcrafted ultrasound descriptors and ConvNeXt embedding dimensions additively contribute to the predicted malignancy score (Tag = 1).

Because ConvNeXt embedding dimensions represent latent deep features and are not directly human-interpretable on a per-dimension basis, their role was assessed through SHAP magnitudes and relative contributions in both global and local explanations. For categorical ultrasound descriptors encoded via label encoding, SHAP values reflect the effect of specific encoded categories rather than a strictly monotonic relationship with the numeric code.

### 3.9. Evaluation Metrics

Model performance was assessed using accuracy, macro-F1, AUC, malignant recall, and benign specificity. Accuracy was defined as the proportion of correctly classified cases. For the macro-F1 score, precision and recall were computed separately for the benign and malignant classes, and the harmonic mean of these values was then averaged across classes to provide a class-balanced measure of performance.

Malignant recall (sensitivity) was defined as the proportion of malignant lesions correctly identified, while benign specificity was defined as the proportion of benign cases correctly classified. Both metrics are clinically relevant because they quantify the model’s ability to avoid false negatives and false positives, respectively. AUC summarizes the trade-off between true-positive rate and false-positive rate across all possible decision thresholds.

Confusion matrices were computed for CV folds and for the external validation set, enabling calculation of all metrics reported in this study.

### 3.10. Computational Complexity and Runtime

The proposed pipeline consists of two stages: feature extraction using a pretrained ConvNeXt-Tiny network and classification using gradient-boosted decision trees. Feature extraction is performed once per image and scales linearly with the number of images. Since ConvNeXt is used in inference mode only, this step incurs a fixed and moderate computational cost. The subsequent CatBoost training operates on a low-dimensional tabular representation and has linear complexity with respect to the number of samples and trees. In practice, both training and inference are computationally lightweight and can be executed efficiently on standard CPU hardware.

### 3.11. Pipeline Flow Diagram

**Figure 2:**
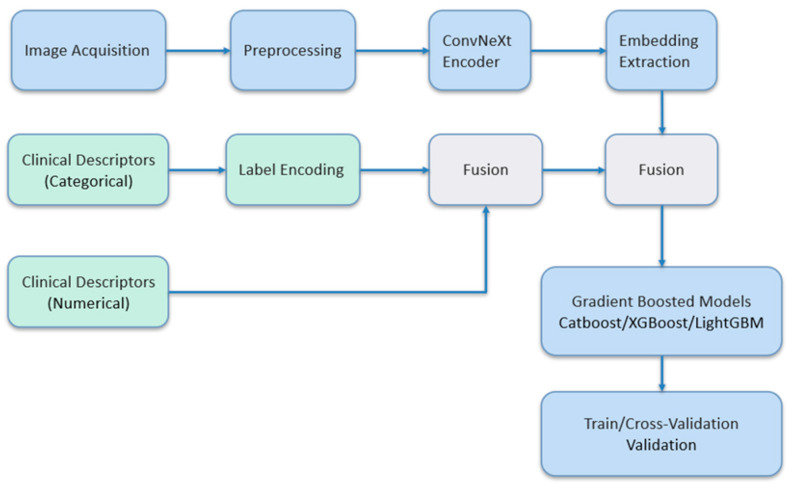
Method overview.

The overall pipeline from image acquisition to evaluation is summarized in Figure 2. Raw US images are preprocessed and fed into a pretrained ConvNeXt encoder to produce embeddings. Sonographic descriptors are encoded using label encoding. The two feature groups are concatenated to produce a tabular matrix that is used to train gradient-boosted models. CV and external validation metrics guide model selection.

## 4. Results

### 4.1. Descriptor-Only Baseline

Using only the encoded sonographic descriptors, all three boosted tree models performed well (Table 2). LightGBM yielded the best external results, achieving 0.88 accuracy and 0.95 AUC, with malignant recall of 0.85 and benign specificity of 0.90.

### 4.2. Hybrid (Embedding+Descriptors)

When ConvNeXt embeddings were concatenated with the descriptors, CV accuracy changed only marginally due to the increased feature dimensionality (Table 3). However, external validation metrics improved: the fused LightGBM model reached 0.88 accuracy, 0.96 AUC, and a malignant recall of 1.00, although benign specificity decreased to 0.80. As shown in Table 3, the hybrid model provides a small but consistent AUC gain over the descriptor-only baseline. Table 4 shows the confusion matrix on the external validation set, illustrating the distribution of correct and incorrect predictions across benign and malignant cases.

### 4.3. Random Feature Subset Search—Top 5 Combinations

The random feature subset search over 500 ten-feature subsets identified several compact combinations that matched or exceeded the performance of the full fused feature set (Table 5). The best subset (Rank 1) combined six descriptors (Calcifications_LE, Halo_LE, Margin_LE, Shape_LE, Skin_thickening_LE, and Symptoms_LE) with four embedding dimensions (emb_117, emb_387, emb_518, and emb_526). Although its CV accuracy (0.817) was slightly lower than that of some other combinations, this subset achieved the highest external accuracy (0.92), the highest AUC (0.9567), and perfect malignant recall (1.00), with only a small number of benign cases misclassified. The variables denoted as emb_xxx correspond to individual dimensions of the 768-dimensional ConvNeXt-Tiny embedding vector. These indices represent latent feature coordinates learned by the pretrained network and do not correspond to any predefined anatomical or semantic descriptors.

### 4.4. Final 10-Feature Subset

**Figure 3:**
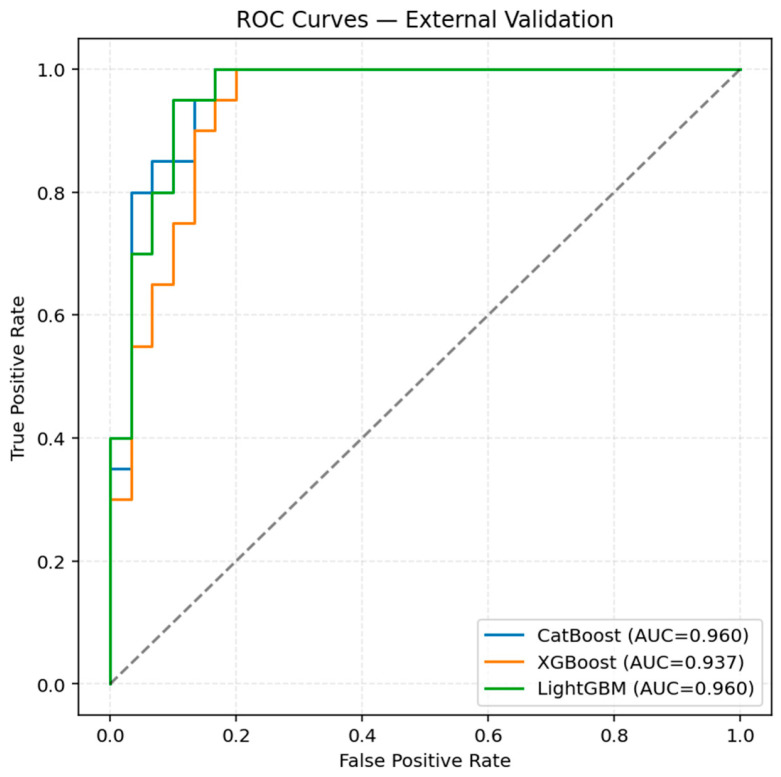
ROC curves over the external validation set.

For the final model, we selected a 10-feature subset that balances performance and clinical interpretability. The chosen features were Age, Calcifications_LE, Halo_LE, emb_239, emb_27, emb_349, emb_419, emb_497, emb_593, and emb_596. On the external validation set, this model achieved an AUC of 0.96 and a malignant recall of 1.00, indicating that all malignant lesions were correctly detected. The inclusion of patient age provides additional clinical context, while the selected embedding dimensions capture discriminative patterns in US texture. The ROC curve of this final model is also included in Figure 3. 

### 4.5. SHAP Analysis

**Figure 4:**
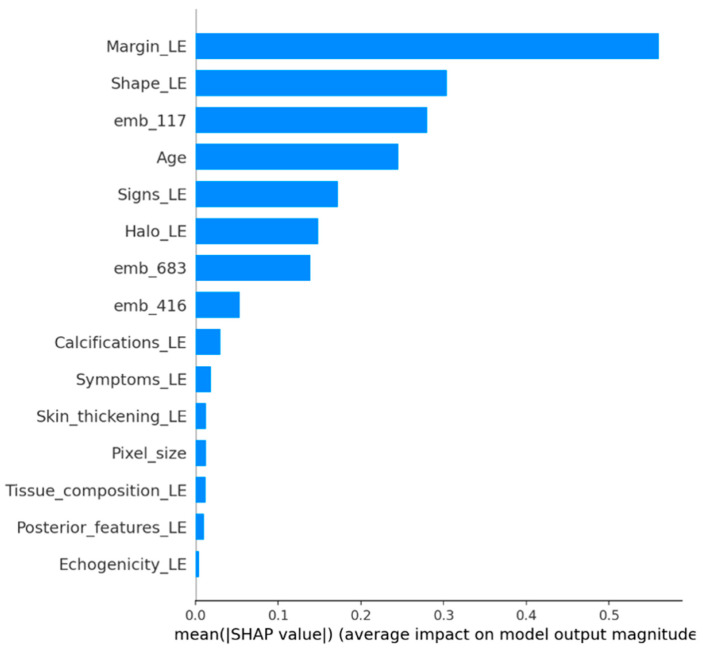
Global SHAP feature importance (mean |SHAP|).

**Figure 5:**
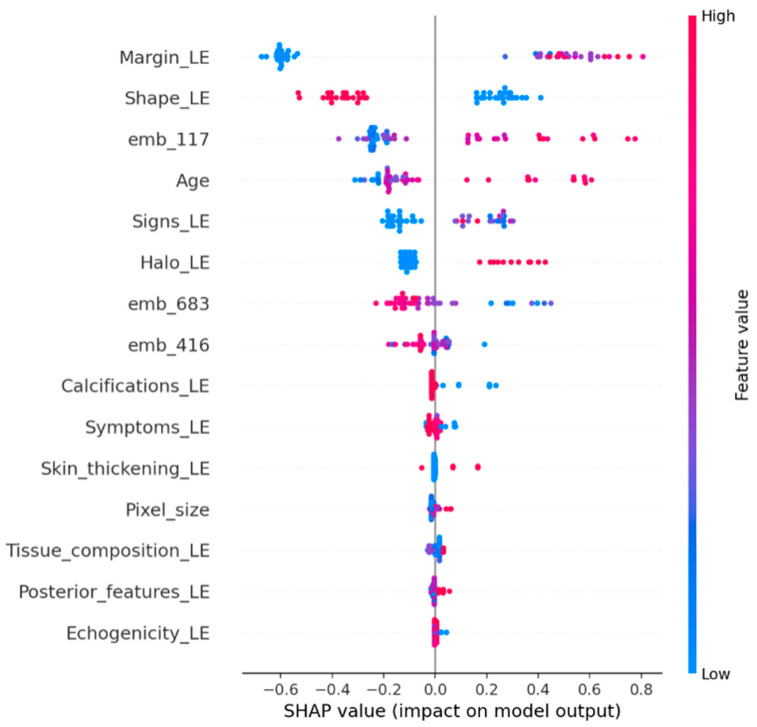
SHAP summary plot showing feature impact distribution.

**Figure 6:**
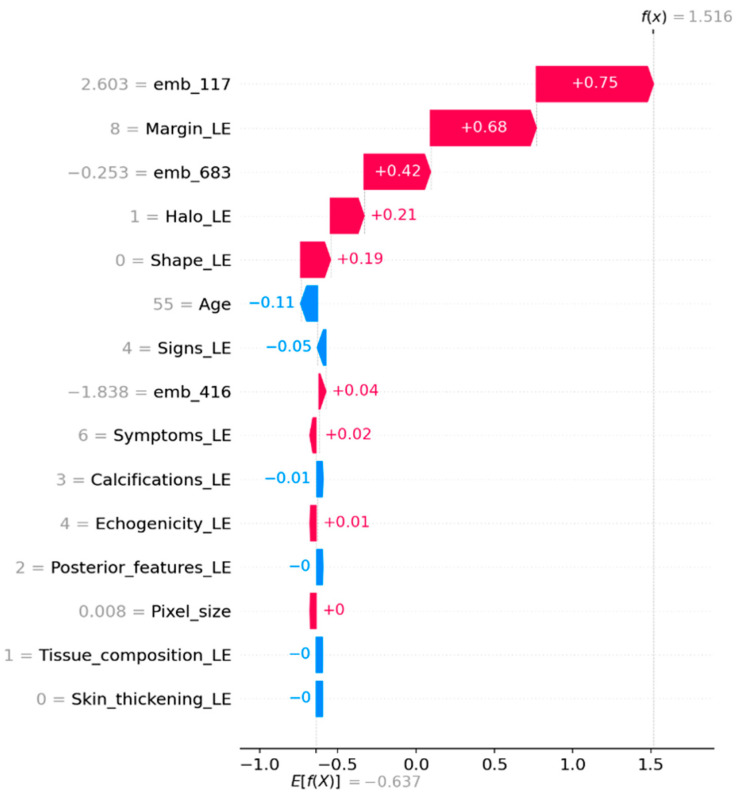
Local SHAP explanation for a malignant case.

**Figure 7:**
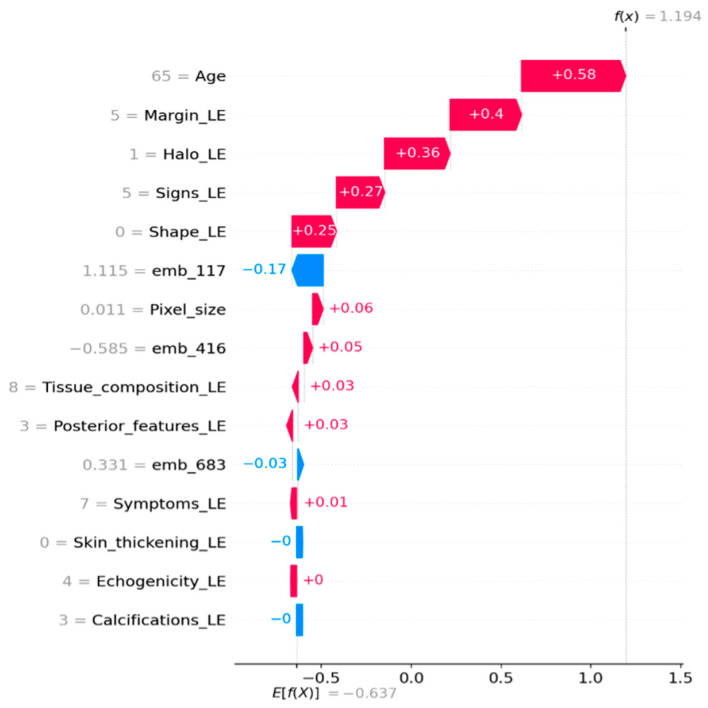
Local SHAP explanation for a representative case.

SHAP analysis was performed on the external validation set to examine feature contributions in the CatBoost hybrid model. Global SHAP importance indicated that margin and shape descriptors were among the strongest contributors to malignant predictions (Figure 4 and Figure 5). In addition, several ConvNeXt embedding dimensions, particularly emb_117, showed substantial contributions. Local SHAP explanations for representative cases further demonstrated that some predictions were driven mainly by embedding features, whereas others were dominated by clinical descriptors (Figure 6 and Figure 7).

## 5. Discussion

This study demonstrates that converting breast US images into a tabular representation enables accurate lesion classification even when only a few hundred cases are available. Instead of training a deep neural network end-to-end—which typically requires large datasets to avoid overfitting—we used a pretrained ConvNeXt Tiny model as a fixed feature extractor and fused its embeddings with clinically recorded sonographic descriptors. This hybrid strategy is motivated by three key considerations:

(i) Breast US datasets are inherently small and heterogeneous, making end-to-end deep learning unreliable in many real-world settings [11,12,17];
(ii) Gradient-boosted trees remain state-of-the-art for tabular data, particularly under limited-sample conditions where they often outperform neural networks [26,27];
(iii) The combination of image-derived features with tabular clinical data, such as health records, has been shown to improve predictive performance [13,15].

The descriptor-only baseline confirmed that boosted trees can already capture substantial diagnostic signal: LightGBM achieved an external AUC of 0.95 using only handcrafted sonographic features. However, augmenting these descriptors with ConvNeXt embeddings improved the ability to detect malignant lesions, increasing the external AUC to 0.96 and achieving a malignant recall of 1.00. The slight reduction in cross-validated accuracy is expected given the increased dimensionality and the modest dataset size, and it underscores the importance of external validation when working with small cohorts.

The random feature subset search further revealed that high performance does not require the full fused feature space. Several compact combinations of six descriptors and four embedding dimensions achieved stronger external validation performance than any full-feature model, including 92% accuracy, an AUC of 0.9567, and perfect malignant recall. These results show that the embeddings contribute complementary information beyond what is encoded in traditional descriptors and that fused feature spaces can be pruned to produce highly interpretable models without sacrificing diagnostic sensitivity. The descriptors selected by the top-performing subsets (e.g., calcifications, halo sign, margin, shape, skin thickening, and symptoms) align with established imaging markers, supporting the clinical plausibility of the model.

In recent years, hybrid approaches that combine deep image features with handcrafted or structured features have been increasingly investigated in medical image analysis. Several studies have shown that fusing CNN-derived representations with conventional features can improve classification performance in various image analysis tasks [28,29,30,31,32]. These studies collectively highlight the potential of hybrid feature representations. More recently, transformer-based and hybrid explainable frameworks have also been proposed for breast cancer diagnosis in mammography and multimodal imaging settings, demonstrating promising performance in both centralized and federated learning scenarios [28,29,30,31]. However, many of them focus on larger datasets, different imaging modalities, or end-to-end deep learning pipelines. In contrast, the present study focuses on small-cohort breast ultrasound data and adopts a tabular fusion framework designed to preserve interpretability while maintaining competitive diagnostic performance. This study shows that integrating abstract deep learning features with intuitive clinical observations allows for high performance without the typical ‘big data’ requirements. We argue that when features are sufficiently informative and multidimensional, a robust classification framework can be established even within the constraints of smaller, real-world clinical cohorts.

To further validate this argument and ensure the model’s clinical grounding, we utilized SHAP analysis on the external validation set. The analysis confirms that margin and shape descriptors are significant contributors to malignant predictions, aligning with established ultrasound assessment criteria (Figure 4 and Figure 5). A small number of ConvNeXt embedding dimensions, especially emb_117, also show significant contributions. This suggests that deep image features capture patterns that handcrafted descriptors do not fully capture. Local SHAP explanations show that some malignant predictions are mostly based on embeddings, while others are mostly based on clinical descriptors (Figure 6 and Figure 7). This supports the strength and mixed nature of the proposed approach.

The final 10-feature model, which includes both age and selected embedding dimensions, balances interpretability, compactness, and performance, making it more suitable for downstream clinical use. Gradient-boosted trees also provide transparent feature importance scores, offering an advantage over deep end-to-end networks whose internal representations are typically opaque.

Despite these strengths, the study has limitations. The dataset is modest in size and originates from a single center, which may limit generalizability across scanners, operators, and populations. Although 500 feature subsets were evaluated, the optimal subset may shift with larger cohorts or with different US acquisition protocols. Future work will therefore focus on validating the method on multi-center clinical datasets and exploring additional imaging modalities such as elastography or Doppler. Beyond US, the general “image-to-tabular” fusion approach presented here may also be applicable to other small-data medical imaging problems, offering a practical and interpretable alternative to end-to-end deep learning.

## 6. Conclusions

This study shows that converting breast US images into tabular form using pretrained ConvNeXt embeddings and subsequently fusing these representations with clinically recorded descriptors enables accurate and robust lesion classification on small datasets. The results demonstrate that image-derived embeddings and structured clinical descriptors provide complementary information: descriptors capture established sonographic markers, while ConvNeXt embeddings encode high-level texture and shape patterns that are difficult to express manually. When combined within a unified tabular representation, these heterogeneous features enable gradient-boosted tree ensembles to achieve strong generalization and clinically meaningful operating points, and subset optimization can identify compact, interpretable feature sets that attain very high malignant recall while controlling false positives.

The use of pretrained deep neural networks as feature extractors has been widely reported to improve the discrimination between benign and malignant breast lesions [14]. By avoiding end-to-end deep learning and instead leveraging pretrained backbones with state-of-the-art tabular learners, the proposed approach mitigates overfitting risks inherent to small US datasets while maintaining transparency and computational efficiency. These characteristics make the method particularly suitable for real-world breast-imaging environments, where annotated datasets are typically small, heterogeneous, and collected under variable acquisition conditions. More broadly, the findings illustrate that hybridizing pretrained embeddings with structured clinical features can yield robust classifiers in limited-sample medical imaging settings, providing a practical and generalizable alternative to end-to-end networks.

As a next step, we intend to further evaluate the proposed multimodal fusion framework using independent clinical ultrasound datasets, with the aim of assessing its robustness across different acquisition conditions and potential inter-institutional variability.

## Figures and Tables

**Table 1:** Dataset composition and class distribution.

Split	Total Images	Malignant (Tag = 1)	Benign (Tag = 0)
Training Set	202	78	124
Hold-out validation set	50	20	30
**Total**	**252**	**98**	**154**

**Table 2:** Performance of models using descriptors only.

Model	CV Accuracy	CV AUC	CV Recall	CV Spec	Val Accuracy	Val AUC	Val Recall	Val Spec
**CatBoost**	0.812	0.9100	0.782	0.831	0.860	0.9533	0.900	0.833
**XGBoost**	0.802	0.8998	0.731	0.847	0.860	0.9400	0.900	0.833
**LightGBM**	0.782	0.8933	0.744	0.807	0.880	0.9500	0.850	0.900

**Table 3:** Performance of models using fused embeddings and descriptors.

Model	CV Accuracy	CV AUC	CV Recall	CV Spec	Val Accuracy	Val AUC	Val Recall	Val Spec
**CatBoost**	0.802	0.9155	0.798	0.837	0.880	0.9600	0.95	0.833
**XGBoost**	0.802	0.9138	0.736	0.859	0.860	0.9367	0.95	0.800
**LightGBM**	0.817	0.9163	0.782	0.851	0.880	0.9600	1.00	0.800

**Table 4:** Confusion matrix on the external validation set (CatBoost, hybrid features).

	Predicted Benign	Predicted Malignant
True Benign	28	2
True Malignant	2	18

**Table 5:** Top five feature subsets from the random search (k = 10).

Rank	Total Score	Features (10 Features)	CV Acc	CV Recall	CV Spec	Val Acc	Val Recall	Val Spec	Val AUC
1	0.9202	Calcifications_LE, Halo_LE, Margin_LE, Shape_LE, Skin_thickening_LE, Symptoms_LE, emb_117, emb_387, emb_518, emb_526	0.817	0.769	0.847	0.920	1.00	0.867	0.9567
2	0.9076	Halo_LE, Margin_LE, Posterior_features_LE, emb_117, emb_137, emb_387, emb_416, emb_497, emb_593, emb_596	0.861	0.821	0.887	0.900	0.95	0.867	0.9433
3	0.8970	Calcifications_LE, Echogenicity_LE, Margin_LE, Shape_LE, Symptoms_LE, emb_239, emb_349, emb_456, emb_549, emb_593	0.856	0.846	0.863	0.860	1.00	0.767	0.8983
4	0.8967	Calcifications_LE, Halo_LE, Margin_LE, Pixel_size, Signs_LE, Symptoms_LE, Tissue_composition_LE, emb_497, emb_518, emb_605	0.837	0.782	0.871	0.900	0.95	0.867	0.9100
5	0.8956	Halo_LE, Margin_LE, Pixel_size, Posterior_features_LE, Shape_LE, Tissue_composition_LE, emb_117, emb_137, emb_201, emb_549	0.847	0.808	0.871	0.900	0.90	0.900	0.960

## Data Availability

The data used in this study are derived from the publicly available BrEaST-Lesions dataset. Any additional data processing outputs generated during the current study are available from the corresponding author upon reasonable request.

## Abbreviations

The following abbreviations are used in this manuscript:

US	Ultrasound
CNNs	Convolutional neural networks
CV	Cross-validation
AUC	Area under the curve
ROC	Receiver operating characteristic
SHAP	SHapley Additive exPlanations
